# Transmission Distortion Affecting Human Noncrossover but Not Crossover Recombination: A Hidden Source of Meiotic Drive

**DOI:** 10.1371/journal.pgen.1004106

**Published:** 2014-02-06

**Authors:** Linda Odenthal-Hesse, Ingrid L. Berg, Amelia Veselis, Alec J. Jeffreys, Celia A. May

**Affiliations:** Department of Genetics, University of Leicester, Leicester, United Kingdom; University of Oxford, United Kingdom

## Abstract

Meiotic recombination ensures the correct segregation of homologous chromosomes during gamete formation and contributes to DNA diversity through both large-scale reciprocal crossovers and very localised gene conversion events, also known as noncrossovers. Considerable progress has been made in understanding factors such as PRDM9 and SNP variants that influence the initiation of recombination at human hotspots but very little is known about factors acting downstream. To address this, we simultaneously analysed both types of recombinant molecule in sperm DNA at six highly active hotspots, and looked for disparity in the transmission of allelic variants indicative of any *cis*-acting influences. At two of the hotspots we identified a novel form of biased transmission that was exclusive to the noncrossover class of recombinant, and which presumably arises through differences between crossovers and noncrossovers in heteroduplex formation and biased mismatch repair. This form of biased gene conversion is not predicted to influence hotspot activity as previously noted for SNPs that affect recombination initiation, but does constitute a powerful and previously undetected source of recombination-driven meiotic drive that by extrapolation may affect thousands of recombination hotspots throughout the human genome. Intriguingly, at both of the hotspots described here, this drive favours strong (G/C) over weak (A/T) base pairs as might be predicted from the well-established correlations between high GC content and recombination activity in mammalian genomes.

## Author Summary

Meiosis is an essential feature of sexual reproduction that maintains chromosome number over generations. This specialised form of cell division creates gametes containing a single copy of each chromosome so that each parent contributes half their genetic information to an offspring. Accurate partitioning first requires intimate association of the two parental copies of each chromosome and concomitant exchange between them. These exchanges consist of both large-scale reciprocal crossovers, essential for correct chromosome segregation, and very localised gene conversion events, or noncrossovers, thought to be involved in correct chromosome pairing. Ordinarily, the reshuffling of genetic variants between generations by recombination will not alter their population frequency only their haplotypic context, with a parent passing on a given variant to 50% of its gametes according to Mendel's law of inheritance. However, by screening for both types of recombinant amongst the sperm DNA of healthy men, we have identified a novel form of biased transmission that is restricted to noncrossovers and favours eventual fixation of one variant over another in the population. This previously undetected source of meiotic drive will not alter recombination propensity but is likely to be a common and potent force acting on the human genome.

## Introduction

During meiosis, homologous chromosomes have to find each other and engage in recombination to segregate accurately. To provoke stable pairing, DNA double-stranded breaks (DSBs) are introduced by SPO11, and subsequently repaired using the homologue instead of the identical sister-chromatid as a template (reviewed in [1]). This tethering of homologous chromosomes provides the necessary connection for accurate reductional segregation. Failure to place at least one crossover (CO) event per chromosome pair can result in non-disjunction and therefore gamete aneuploidy, a major cause of developmental abnormality and spontaneous miscarriage in humans [2].

Recombination events are not randomly distributed in the human genome, but instead cluster into “hotspots” 1–2 kb wide in which crossover resolution points define a normal distribution around a central point, presumably reflecting a diffuse zone of DSB formation plus the outward migration of Holliday junction intermediates from a DSB site [3], [4]. Hotspot activity is regulated in *trans* by the protein “PR-domain containing 9” (PRDM9) [5]–[9], whose DNA binding domain consists of tandem-repeat zinc-fingers (ZnFs) encoded by a minisatellite. Small variations in this tandem array can alter the DNA binding specificity of PRDM9 as shown by significant differences in hotspot usage between individuals with differing alleles [5], [6], [9]–[11]. PRDM9 might function by triggering chromatin remodelling via histone 3 lysine 4 trimethylation, allowing SPO11 to introduce DSBs and thus initiate recombination [12], [13].

Additional insights into factors influencing human hotspot activity have come through observations of biased gene conversion accompanying CO. Extensive studies in yeast have indicated that DSB formation is followed by 5′ resection, invasion of the resulting 3′-overhang into a non-sister chromatid, D-loop formation and DNA synthesis, with the repair of heteroduplex DNA leading to gene conversion [14], [15]. Ordinarily, there is no net bias in this process as CO resolution points from reciprocal orientations map to the same intervals in humans [16], [17]. However, biased conversions accompanying CO have been directly observed in some sperm assays as deviations from the expected 1∶1 ratio of allele frequencies amongst recombinant progeny [9], [18]–[20]. This can arise if initiation occurs more frequently on one chromosome than the other and the broken strand is corrected with information from the uncut homologue.

Heterozygosity at a single SNP can lead to such differences in initiation between interacting haplotypes and can directly influence hotspot activity in *cis* [18], [19]. Indeed the over-transmission of alleles from a DSB-suppressed haplotype to recombinant progeny results in meiotic drive in favour of hotspot-suppressing variants and therefore hotspot extinction over time; the stronger the meiotic drive, the faster the hotspot will attenuate [18], [19], [21], [22]. This form of *cis*-regulation has so far been described at four human recombination hotspots, the autosomal hotspots *DNA2*, *NID1* [18], [19] and 5A [9] as well as hotspot *SPRY3* located in the minor pseudoautosomal region (PAR2) [20]. In several cases suppressing SNP variants have been noted to occur within degenerate GC-rich sequences proposed as binding sites for different classes of *PRDM9* variants [5], [8], [9], [11], [20], [23], [24]. However hotspots with and without these sequence motifs can be equally regulated by PRDM9 and motif-disrupting SNPs have also been associated with active haplotypes [9]. Indeed, recent *in vitro* studies have highlighted the complexity with which the long ZnF array of the mouse Prdm9 protein binds to DNA [25] and this is likely true of human PRDM9 too.

Recombination hotspot analysis in humans has to date largely focused on the description of COs as these can be readily detected in pools of sperm DNA even in the presence of a large excess of non-recombined molecules [26]. However in the mouse, cytological evidence has shown that only ∼10% of DSBs go on to form COs while the remainder are repaired without exchange of flanking markers [27], [28]. These localised gene conversion-only events are also known as noncrossovers (NCOs) and are only detectable if heteroduplex DNA intermediates encompass informative markers. NCOs do not appear to be sufficient for correct chromosome segregation but are thought to aid homologue pairing and/or the regulated placement of COs [29].

SNP enrichment techniques have allowed the identification of NCO events at human recombination hotspots [30] and have shown that these gene conversions co-localise with the centre of the CO distribution and involve short tracts [31]. Given that the same SNP heterozygosities that lead to transmission distortion (TD) amongst COs similarly affect NCOs [19], and that NCO frequency as well as CO frequency is regulated by PRDM9 [9], [20], it is clear that these two classes of recombinants arise from the same initiating DSBs. Indeed, yeast models postulate distinct pathways that diverge shortly after DSB initiation, with the canonical DSB repair (DSBR) pathway [32] mainly producing COs and the synthesis-dependent strand annealing (SDSA) pathway [33], [34] mainly NCOs [35].

We have little information on the factors influencing human recombination events downstream of DSB induction. However, it is clear from sperm analysis that the balance between NCO and CO can be very variable between hotspots (<1∶12 to ∼3∶1 NCO∶CO) [36], and at least at *SPRY3* between men too (∼35-fold range) [20]. At the *SPRY3* hotspot some men also show more extreme TD amongst NCOs than COs at a hotspot-attenuating SNP, suggesting that *cis*-effects are mediated not only through recombination initiation frequencies but also through downstream processing. Given that this hotspot is located in the pseudoautosomal region PAR2, it is unclear whether this finding is more generally applicable to hotspots elsewhere in the genome.

To gain further insight into differential processing, we have therefore analysed six highly active and marker-rich autosomal hotspots for both COs and NCOs. At two of these hotspots, we identified a novel type of biased gene conversion acting solely upon the NCO class of recombinant. This bias is not predicted to lead to hotspot attenuation but represents a previously unappreciated yet significant source of meiotic drive operating in the human genome.

## Results

### Detection of CO and NCO Recombinants at Six Human Hotspots

We initially surveyed six autosomal recombination hotspots using an assay capable of efficiently detecting COs and NCOs simultaneously in sperm DNA [26]. Hotspots were selected for their intense CO activities (sperm recombination frequencies ranging 0.13–1.10%) [5], [37], coupled with at least one common SNP within ±150 bp of the centre of the hotspot to facilitate detection of NCOs (median SNP number = 2, median minor allele frequency = 0.346, median distance from centre = 29 bp, see also Table S1). Five of the chosen hotspots (E, F, H, K, T) are activated by the common A variant of PRDM9 [5](AJ Jeffreys, *unpublished data*), while one (hotspot 5A) is activated by PRDM9 C and C-related variants [9].

For each hotspot, we analysed reciprocal COs and NCOs in two men and tested whether any markers showed transmission distortion (TD) in recombinants (Table S2). Combining data for each marker across the two men at hotspots E, H and T failed to reveal any significant TD, though at hotspot H, CO distortion occurred at marker H7.6 (rs3899614) located 102 bp from the centre of the hotspot albeit in just one of the two men tested. In this instance crossover breakpoints from opposite orientations were found to map to intervals displaced by 195 bp with TD in favour of G over A at this marker (71% *vs.* 29%, respectively; *P* = 0.029, two-tailed exact binomial). However, NCO counts for this man were low (6 in favour of H7.6G and 4 in favour of H7.6A), compatible with both TD of the same strength as seen in COs (*P* = 0.70, Fisher's exact test) but also with no TD at all amongst NCOs (*P* = 0.75, two-tailed exact binomial).

This preliminary screen did reveal TD in both CO and NCO for hotspot 5A. One of the two men analysed was heterozygous for SNP 5A7.2 (rs116141470), the closest marker at just 21 bp from the centre of this hotspot. In this case, a shift of 237 bp was noted between CO breakpoints from the two orientations indicative of TD, and overtransmission of the G allele relative to the A allele was noted for both types of recombinant. Although the TD seen for NCOs (83∶17) was somewhat stronger than that for COs (67∶33), this difference was not significant (*P* = 0.09, Fisher's exact test). A more extensive survey of recombination at this PRDM9 C-regulated hotspot has subsequently substantiated the finding that heterozygosity at 5A7.2 drives this biased gene conversion [9]. The TD seen at hotspot 5A is entirely consistent with differential initiation of recombination between haplotypes, a phenomenon that has been noted at other hotspots [18]–[21].

Much more unusually, we observed incidences of significant TD that seemed to be exclusive to the NCO class of recombinant at hotspots F and K. At each of these hotspots, strong TD was seen at a single site of SNP heterozygosity close to the centre of the hotspot, while additional NCOs that did not span this marker did not show significantly biased TD. To explore this phenomenon we extended the panel of men analysed for each of hotspots F and K (Table S3).

### High-Resolution Crossover Analysis

We detected 1,028 COs in over 129,000 sperm DNA molecules screened across 10 men at hotspot F, as well as 599 COs in over 247,000 molecules screened across 13 men at hotspot K. The ranges of CO frequencies obtained in our assays were comparable to those observed by conventional repulsion-phase sperm CO assays for men homozygous for the activating A variant of PRDM9 [5], varying ∼6-fold between men at hotspot F and ∼11-fold at hotspot K. Reciprocal CO products arose with the same frequency in all assays, and pooled CO exchange points from all men displayed quasi-normal distributions, estimated to be 1.60 kb wide at hotspot F and 1.46 kb wide at hotspot K. The widths and locations of hotspot centres from these distributions were similar to those obtained using crossover breakpoint mapping following conventional sperm CO assays at the same hotspots [37], establishing that our assays were providing reliable data (Table S4).

### Morphology of Gene Conversions Gradients at Hotspots F and K

**Figure 1:**
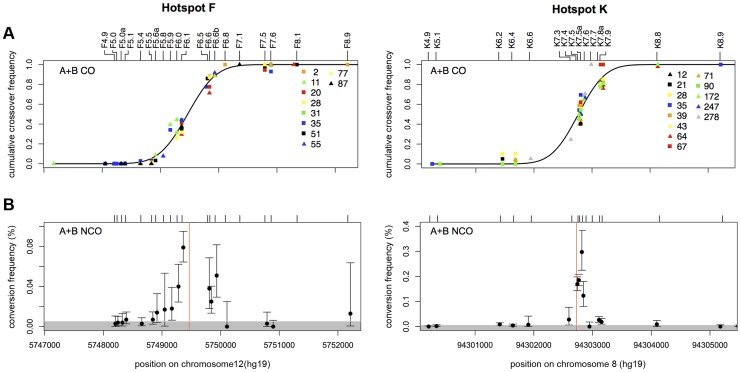
CO and NCO distributions at recombination hotspots F and K. (A) Combined reciprocal cumulative CO distributions. SNP markers across the assay intervals are indicated by tick marks and local names on the top of the box plots (see Table S6 for dbSNP identifiers). Data points represent the observed cumulative proportion of COs pooled from reciprocal assays (A+B COs) at each informative marker for a given man. Different coloured symbols represent different men that are numbered as in refs 5 and 9. A total of 1028 COs were characterised from 10 men at hotspot F (mean CO frequency per sperm 0.81±0.41%) and 599 COs from 13 men at hotspot K (mean CO frequency per sperm 0.26±0.1%). A black line shows the best-fit cumulative CO distribution for each hotspot. (B) NCO gene conversion frequency per SNP, averaged over reciprocal assays (A+B NCOs). Mean conversion frequencies were determined by Poisson-approximation with 95% confidence intervals estimated by simulation. The grey shaded area marks what appears to be a background zone of presumably PCR mis-incorporation that results in false-positive single-SNP NCO artefacts that arise at a frequency of one per ∼15000 progenitor molecules tested. Hotspot centres, as defined by CO distributions, are indicated by red lines. Individual graphs showing the NCO gene conversion frequencies for each man in each orientation can be seen in Figures S1, S2, S3.

Only NCOs that span at least one informative SNP site and lead to gene conversion are detectable. Hotspots F and K have good informative SNP densities close to the hotspot centres (Table S1), allowing us to identify 153 pools of sperm DNA containing at least one NCO molecule at hotspot F and 667 pools at hotspot K. Most NCO events involved conversion of a single SNP (92% at hotspot F and 75% at hotspot K), though the ability to detect co-conversion is heavily reliant on the location of heterozygous markers carried by a given man. In both cases, the conversion gradients appeared symmetrically distributed around the hotspot centre consistent with recombination being initiated not at a point, but rather a zone within the recombination hotspot (Figure 1) [31]. Although most NCOs involved only the central markers, a number of flanking heterozygosities, up to ∼550 bp from the centre of hotspot F and ∼130 bp from the centre of hotspot K, were also involved in apparent NCO gene conversion. This mapping of NCOs throughout the hotspot, with a peak at the centre has also been observed at mouse hotspot A3 suggesting secondary sites of lower DSB formation within the hotspot interval [28]. Thus, the general morphology of these conversion gradients did not appear to be exceptional.

### Biased Gene Conversion in NCOs but Not COs

**Figure 2:**
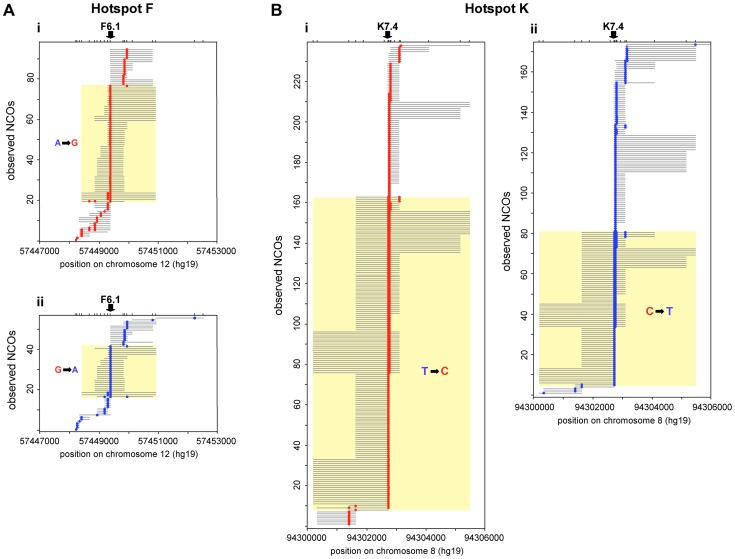
Transmission distortion specifically in NCOs. NCO recombinants detected in reciprocal recombination assays for 10 men heterozygous at F6.1 in hotspot F (A), and 8 men heterozygous at K7.4 in hotspot K (B). (i) Assays with selector sites in phase with the parental haplotype carrying the under-transmitted allele (F6.1A or K7.4T, respectively) and thus with recombinants carrying marker(s) from the haplotype bearing the over-transmitted allele (F6.1G or K7.4C respectively) are shown in red. (ii) Opposite phase recombinants are shown in blue. The structure of each observed NCO is shown separately, with converted markers indicated by coloured circles and maximum conversion tract lengths shown by grey lines. NCOs that span the key markers (arrowed) are indicated by yellow boxes; significantly more of these events are seen in (i) than in (ii) (hotspot F: *P* = 0.00016; hotspot K: *P* = 3.0×10^−7^; two-tailed exact binomial tests). Events outside the yellow boxes that do not encompass the key markers, show no disparity in frequency between orientations (i) and (ii) (hotspot F: *P* = 0.457; hotspot K: *P* = 0.295; two-tailed exact binomial tests). Note that tract length estimation is difficult because minimal conversion tracts that include only the converted markers almost certainly underestimate the true conversion tract length while maximal lengths, which correspond to the entire interval between flanking non-converted markers, can be heavily influenced by marker distribution. The minimum conversion tract length for any single site event is clearly 1 bp whilst for multisite events it was 83 bp at hotspot F and 25 bp at hotspot K. However, the maximum lengths of ranged from 118 bp to 2504 bp for hotspot F and 77 bp to 4970 bp for hotspot K.

COs and NCOs were separately tested for disparity in reciprocal conversion frequency at each marker across the hotspot interval [19]. At both hotspots we detected a greater number of NCOs in one orientation compared to the other and noted that this phenomenon was focused around one, nearly-central, SNP at each hotspot (Figure 2).

At hotspot F the highest conversion frequency was observed at marker F6.1A/G (rs10492181). Here all ten F6.1A/G-heterozygous men tested exhibited more F6.1A→G than F6.1G→A conversions, so extreme in man 28 that all conversions were of the F6.1A→G type. On average, 71% of NCOs spanning F6.1 contained the G rather than the A allele (Table 1), a ratio significantly different from 50∶50 (*P* = 0.00016, two-tailed exact binomial). In contrast, additional NCOs that did not include F6.1 did not show this bias, with only 53% of NCOs involving transfer of markers from the F6.1G haplotype (*P* = 0.457, two-tailed exact binomial) (Table 2). Finally, these men did not show any evidence for TD amongst their COs, with only 51% of such events containing the G allele, a transmission ratio not significantly different from 50% (*P* = 0.480, two-tailed exact binomial) (Table 1). The mean transmission ratio in COs and NCOs (51% *vs.* 71%) is significantly different (*P* = 0.0006, χ^2^ test, 1d.f.), indicating that TD is specifically affecting F6.1 only in NCOs.

**Table 1:** Transmission at SNPs showing biased gene conversion in NCOs at hotspots F and K.

Hotspot F		COs:			NCOs:		
man	molecules screened	with F6.1G	with F6.1A	% carrying F6.1G	with F6.1G	with F6.1A	% carrying F6.1G
28	16660	74	94	44	13	0	100
35	12218	12	13	48	5	1	83
77	11960	32	33	49	4	1	80
87	11935	42	32	57	3	1	75
55	11960	33	42	44	7	3	70
11	16560	26	18	59	6	3	67
20	12108	58	75	44	4	2	67
31	11785	81	70	54	12	6	67
51	12016	77	60	56	10	7	59
2	11960	94	62	60	6	5	55
	TOTAL	529	499	51	70	29	71

Hotspot K		COs:			NCOs:		
man	molecules screened	with K7.4C	with K7.4T	% carrying K7.4C	with K7.4C	with K7.4T	% carrying K7.4C
39	16610	16	15	52	21	6	78
12	15912	22	20	52	26	8	76
35	29440	7	5	58	15	6	71
43	16354	18	36	33	17	7	71
90	29440	35	26	57	28	12	70
71	16503	39	33	54	26	12	68
28	13800	6	14	30	12	8	60
172	15906	16	20	44	17	18	49
	TOTAL	159	169	48	162	77	68

Men are numbered as in refs 5, 9 & 37. The combined number of molecules screened in the two orientations is shown for each man. Poisson-corrected numbers of recombinants are rounded up to the next integer; this Poisson-correction was modest, with observed CO numbers increased by a factor of 1.17 and 1.03 for hotspots F and K respectively, and NCO numbers being increased by a factor of 1.14 and 1.06 respectively. There is no evidence for heterogeneity between individuals in the strength of TD in NCOs in favour of F6.1G or K7.4C (hotspot F: *P* = 0.83, 2×10 contingency table, 9 d.f.; hotspot K: *P* = 0.24, 2×8 contingency table, 7 d.f.). Marginally significant variation between individuals was noted for COs at hotspot F (*P* = 0.047, 2×10 contingency table, 9 d.f.) but no significant variation for this class of recombinant amongst the men screened at hotspot K (*P* = 0.11, 2×8 contingency table, 7 d.f.).

**Table 2:** Transmission into NCOs at additional SNPs at hotspots F and K shows no conversion bias.

Hotspot F				
man	additional NCOs^1^	from F6.1G haplotype	from F6.1A haplotype	% from F6.1G haplotype
28	4	4	0	100
35	4	3	1	75
51	10	7	3	70
55	10	6	4	60
11	16	8	8	50
77	6	3	3	50
20	9	4	5	44
2	10	4	6	40
31	6	1	5	17
87	0	0	0	-
TOTAL	75	40	35	53

Hotspot K				
man	additional NCOs^1^	from K7.4C haplotype	from K7.4T haplotype	% from K7.4C haplotype
35	5	3	2	60
90	31	18	13	58
71	27	15	12	56
28	17	8	9	47
12	20	9	11	45
172	65	27	38	42
39	19	8	11	42
43	12	4	8	33
TOTAL	196	92	104	47

1 *i.e.* excluding any NCO event that involves markers F6.1 or K7.4, including co-conversion events.

A very similar phenomenon was observed at hotspot K. Eight men heterozygous for marker K7.4C/T (rs1374633) all showed directionality in NCOs, with 68% of events having acquired the C-allele (Table 1), a highly significant deviation from the expected 50∶50 transmission ratio (*P* = 3.0×10^−7^, two-tailed exact binomial). Marker K7.5 just 24 bp away, showed a weaker degree of transmission bias in NCOs, probably as a consequence of co-conversion with K7.4 (58% of alleles carried on the same haplotype as K7.4C were transmitted to NCO progeny, *P* = 0.013, two-tailed exact binomial, with transmission bias independent of K7.5 allele status). Indeed as observed at hotspot F, NCOs that did not span K7.4 did not show significant departure from parity (47% contained markers from the K7.4C haplotype, *P* = 0.295, two-tailed exact binomial) (Figure 2 and Table 2). Equally there was no indication of significant TD when COs were considered collectively (48% contained K7.4C, *P* = 0.869, two-tailed exact binomial). This again suggests that NCOs but not COs are affected by strong TD at this hotspot, but only if they include the central marker K7.4C/T.

**Figure 3:**
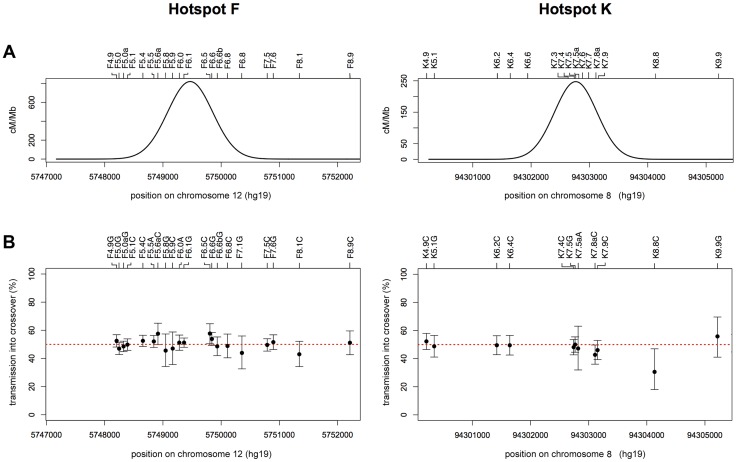
No evidence for CO asymmetry. (A) Least-squares best-fit normal distribution of reciprocal A+B COs for all men combined at hotspot F (left) and hotspot K (right). (B) Transmission frequencies of SNP alleles into reciprocal A+B COs, with 95% CIs calculated by Bayesian analysis. Transmission of the strong allele (C or G) is shown for transition polymorphisms, and transmission of the purine allele is shown (A or G) for transversion polymorphisms. Data for the hotspot F region are derived from all 10 men analysed since they are all heterozygous at SNP F6.1 (left), whilst those for the hotspot K region are from the 8 men heterozygous for SNP K7.4 (right). The two alleles at each of the 20 markers in and around hotspot F show parity in transmission to COs as determined by two-tailed exact binomial tests (all *P* values>0.05, without Bonferroni correction). Of the 11 markers analysable in the hotspot K region, only the alleles at K8.8 showed deviation from 50% transmission (*P* = 0.029, two-tailed exact binomial). The disparity at this marker, which is located outside of the hotspot and informative in just 1 man, is not significant if a Bonferroni correction is applied (*P* = 0.319).

As haplotypes differ between men, and the alleles at these central SNPs are on different backgrounds, then the biases we have seen in NCOs at hotspots F and K are most likely triggered by the affected markers themselves. We have previously identified *cis*-acting effects responsible for transmission bias equally affecting both COs and NCOs as a result of differences in recombination initiation frequency between haplotypes [9], [19], [20]. However, we saw no difference in recombination frequencies in the five men homozygous for either the T or C allele at K7.4, with T/T and C/C homozygotes showing indistinguishable crossover frequencies (0.29% and 0.30% respectively, *P* = 0.74, **χ^2^** test, 1d.f.) and thus no evidence for reduced COs in men homozygous for the over-transmitted C allele. This is consistent with the lack of TD in crossovers at these hotspots (Figure 3) and suggests that TD in NCOs is intimately linked with heterozygosity at these central SNPs.

### Balance of NCOs to COs amongst F6.1 and K7.4 Heterozygotes

Comparisons of the ratio of NCOs spanning the key SNPs with COs could be made for the 10 men heterozygous for F6.1 and the 8 men heterozygous at K7.4. At both hotspots, CO and NCO frequencies were positively correlated (hotspot F: *P* = 0.03, Pearson's *r* = 0.67; hotspot K: *P* = 0.03, Pearson's *r* = 0.75). In contrast to observations of quite extreme variation in NCO∶CO ratios between men (35-fold) at the PAR2 hotspot *SPRY3* [20], more modest 5-fold inter-individual variation was seen at hotspot F and 4-fold at hotspot K (hotspot F: *P* = 0.328, 2×10 contingency table, 9 d.f.; hotspot K: *P* = 0.08, 2×8 contingency table, 7 d.f., with Bonferroni correction) (Table 3). The data are therefore consistent with a constant NCO∶CO ratio across men of ∼1∶11 at hotspot F and 1∶1.3 at hotspot K, with the differences between the hotspots most likely reflecting the relative locations of F6.1 and K7.4 from their respective hotspot centres (92 bp and 15 bp respectively).

**Table 3:** NCO to CO ratio at the driven SNP for hotspots F and K.

Hotspot F			
man	CO frequency, %	NCO frequency at F6.1, %	NCO∶CO ratio
20	1.098	0.050	1∶22.2
87	0.620	0.034	1∶18.5
2	1.304	0.092	1∶14.2
77	0.543	0.042	1∶13.0
28	1.008	0.078	1∶12.9
31	1.281	0.153	1∶8.4
51	1.140	0.141	1∶8.1
55	0.627	0.084	1∶7.5
11	0.266	0.054	1∶4.9
35	0.205	0.049	1∶4.2
		MEAN	∼1∶11
			5.3-fold range

Hotspot K			
man	CO frequency, %	NCO frequency at K7.4, %	NCO∶CO ratio
43	0.330	0.147	1∶2.25
71	0.436	0.230	1∶1.89
90	0.207	0.136	1∶1.53
12	0.264	0.214	1∶1.24
39	0.187	0.163	1∶1.15
172	0.226	0.220	1∶1.03
28	0.145	0.145	1∶1
35	0.041	0.071	1∶0.57
		MEAN	∼1∶1.3
			3.9-fold range

### Impact of Meiotic Drive in NCO Events

The strength of meiotic drive at the population level is governed by the NCO frequency at the driven SNP as well as the degree of TD. At hotspot F, SNP F6.1 converts at an average frequency of 0.08% per sperm and displays TD to a degree of 71∶29 in favour of the G-allele. Together these will give gametic ratios in heterozygotes of 50.0167∶49.9833. Although this ratio is very close to 50∶50, population simulations show that this meiotic drive does have an effect, increasing the likelihood of eventual fixation of the driven G-allele from 14% (its current population frequency in Europeans) to 85% and with fixation occurring somewhat faster than for a non-driven allele (in ∼300,000 years, about 40% of the time required for fixation of the G-allele in absence of meiotic drive). Similarly at hotspot K, drive at marker K7.4 is determined by a NCO frequency of 0.16% and a ratio of 68∶32 in favour of the C-allele, a strength that virtually guarantees eventual fixation of the C-allele, increasing from 73% without drive to >99.8% with drive, and with fixation occurring in only ∼95,000 years, about 24% of the time required in the absence of meiotic drive.

## Discussion

We describe for the first time direct evidence of TD arising solely from the NCO class of recombinant at two human hotspots. In each case, a single SNP heterozygosity close to the centre of the hotspot appears to be triggering this biased gene conversion but without effect on *de novo* CO events. Thus, hotspots that appear to be generating balanced recombinant progeny when analysed just for COs may in fact harbour biased gene conversion generated through the production of NCOs.

Until now, biased gene conversion at human hotspots has predominantly been observed amongst COs, the more widely studied class of recombinant [18], [19], [21]. Where NCOs have also been studied at these hotspots, a comparable bias has been noted amongst this class of event too [9], [19], [20]. At such hotspots, haplotypes can be designated as being either active or relatively suppressed, since men homozygous for the opposing alleles at the key SNPs display substantially different recombination frequencies. This is consistent with differential initiation between haplotypes causing biased transmission of SNP alleles. The ensuing TD leads to so-called hotspot drive in favour of hotspot-suppressing variants [38], which along with the rapid evolution of PRDM9 [39]–[41] is likely responsible for the fast turnover in hotspot locations [8], [21], [42]–[45].

In contrast, the biased conversion noted at hotspots F and K in the present study was restricted to NCOs, and a model of preferential initiation cannot easily explain this. Indeed, men homozygous for different alleles at the key SNP (K7.4) at hotspot K display very similar CO frequencies, indicating that DSBs are induced with comparable frequencies on both chromosomes. These data therefore suggest that the biases in NCO at hotspots F and K arise at a later stage and that the intermediates destined for the two fates must be recognised or processed differently during mismatch repair (MMR).

Under a classical model of MMR, wherein mismatches are deeply embedded in a patch of heteroduplex, a bias could occur if there is a strand preference for introducing a nick since this determines which of the two DNA strands is degraded and thus ultimately establishes the direction of repair [46]. In this case, the nicking preference would be directly dictated by the two bases involved in the mismatch. Alternatively, if the mismatch occurs during strand invasion, then a bias might arise if the efficiency of mismatch recognition depends on the orientation of the mismatched bases with respect to the invading and recipient strands. Removal of mismatches at this stage is achieved through chew-back of the invading strand followed by repair synthesis using the recipient strand as a template [15]. There are a number of possibilities as to why such biased MMR should preferentially affect NCOs and not COs. For example, it could be that the chromatin configurations for the two types of intermediate differ, with those destined to be NCOs being more open and thus allowing better access for the MMR machinery. Another explanation might be that NCO invasions persist for a longer time, allowing biased MMR to occur, while CO invasions are processed more rapidly, healing the DNA ends and thus preventing biased free-end-mediated MMR from acting. Alternatively, it could also be that very short invasions lead to NCOs and that any mismatches within these heteroduplex tracts are easily recognised by the inherently biased MMR system. In contrast, COs might involve longer invasions that might more successfully hide such mismatches from this process.

Our data from these two hotspots represent the largest autosomal surveys of inter-individual variation in NCO∶CO ratio to date. The only comparable dataset is that from the PAR2 hotspot *SPRY3*, for which 35-fold variation between men was noted [20]. This is not dissimilar to the difference previously observed between hotspots as estimated from just one or two men at each. However, at each of hotspots F and K the observed differences in this ratio are much more modest and not significantly different between men, supporting the possibility that variability of the NCO∶CO ratio at *SPRY3* may reflect aspects of chromosome pairing and exchange that are unique to the sex chromosomes in male meiosis, rather than being a more general feature of hotspots [20].

We have observed this biased NCO conversion at two recombination hotspots from a total of six examined. This relatively high proportion suggests that NCO-specific bias is likely to be a general phenomenon, potentially affecting thousands of the 33,000 human recombination hotspots identified from LD patterns in the human genome [47]. In contrast to the previously described instances of hotspot drive, this form of biased gene conversion will not lead to the demise of recombination hotspots, though it may nonetheless have a significant impact on the base composition of the genome. Interestingly, at both F and K, the affected SNPs in NCOs display preferential repair of weak (A/T) to strong (G/C) base pairs. Genomes are known to have evolved towards greater GC richness [48], with the enrichment of GC alleles corresponding to sites of recombination [49], [50], yet our empirical studies of TD that affects both CO and NCO in humans have thus far failed to show any overriding base composition bias [9], [18]–[21]. Of course it remains to be seen whether the GC bias seen at F and K also occurs at other hotspots that might exhibit NCO-specific biased gene conversion. We have shown that this form of meiotic drive is sufficiently strong to promote allele fixation, and it is tempting to speculate that our findings might represent a form of biased gene conversion acting on the human genome that contributes to the correlation between GC content and recombination and thus evolution of genomic base composition.

## Materials and Methods

### Ethics Statement

Semen samples were collected with informed consent and approval from the Leicestershire Health Authority Research Ethics Committee (ref 6659).

### Sample Preparation

Sperm DNA was prepared as described previously [16] and quantified on a NanoDrop1000 spectrophotometer. For details of donors, see [5], [9].

### Hotspot Selection

Autosomal hotspots were chosen amongst those already characterised in Leicester by sperm CO assays [37], [5], [9] on the basis of both CO activity and SNP density over the centre of the hotspot. Intense CO activity (*i.e.* sperm CO frequency >0.1%) allowed sufficient numbers of recombinants to be detected efficiently. Since previous analysis of NCOs had indicated that conversion tracts associated with such events were most likely to be on average somewhere between 55 and 290 bp long and centred around the peak of CO activity [31], hotspots were also chosen on the basis of having at least one SNP within ±150 bp of the predicted centre of the hotspot and with a minor allele frequency >0.2. The latter criterion was applied to maximise the number of analysable semen donors. See Table S1 for specific details of each hotspot analysed.

### SNP Genotyping

Routine genotyping was performed on whole-genome amplified DNA, generated from 40 ng aliquots of each DNA using the GenomiPhi HY DNA amplification kit (GE Healthcare Bio-Sciences). Hotspot target regions were amplified in several partially overlapping PCRs by successive rounds of nested PCR, the products transferred onto nylon membranes and genotyped by allele-specific oligonucleotide (ASO) hybridization as described previously [26], [37], [9]. The linkage phase of internal SNPs was determined by testing separated haplotypes by sequential ASO hybridization at each of the heterozygous SNPs.

### Additional Sequence Polymorphisms

High SNP densities allowed for more accurate crossover breakpoint mapping and conversion tract length estimation. The hotspot centre region was therefore re-sequenced in men chosen for analysis, to identify any additional SNPs. Parental haplotypes of test individuals were separately amplified using allele-specific primers directed to markers outside of the hotspot interval. Excess primer and unincorporated dNTPs were removed using 1.4 U/µl exonuclease I (New England Biolabs) and 0.21 U/µl shrimp alkaline phosphatase (Roche), with incubation at 37 °C for 60 min, followed by 15 min at 80 °C. Universal sequencing primers were designed for 3–4 targets covering the hotspot, with targets overlapping by 100 bp to ensure complete coverage. Standard 20 µl Big Dye Terminator v 3.1 sequencing reactions were carried out, the extension products purified using Performa DTR-gel filtration Cartridges and then separated on a 3730 DNA Analyser (Applied Biosystems).

### Detecting Sperm Recombinants

Hotspot intervals were selectively amplified from small pools of sperm DNA, each containing between 15 and 45 amplifiable molecules, *i.e.* assuming a 50% amplification efficiency for single DNA molecules in long-range allele-specific PCR [26]. Two nested sets of allele-specific primers (ASPs), which flank only one side of the hotspot, together with universal primers on the other side of the hotspot, were used to selectively amplify one haplotype at a time, as described in [26]. PCR reactions were carried out in the PCR-buffer described elsewhere [51], supplemented with 12.5 mM Tris-base (ultra-grade for molecular biology, Fluka Chemie, Buchs, Switzerland), 0.2 µM each of forward and reverse primer, 0.025 U/µl *Taq*-Polymerase plus 0.0033 U/µl *Pfu*-Polymerase and 0.5 µg/ml carrier salmon sperm DNA (Sigma-Aldrich, Gillingham, UK). Haplotype-specific PCR products were dot-blotted onto nylon membranes and then probed for the presence of recombined markers by hybridisation with ^32^P-labelled ASOs specific to the non-selected haplotype, using the TMAC method [26]. See Tables S3, S5 and S6 for full details of primer sequences, PCR conditions and ASO probes used for hotspots F and K.

### Meiotic Drive Simulations

The chance of allele fixation and mean time to fixation were determined for two scenarios, firstly for no meiotic drive and secondly, using the observed level of transmission distortion on the driven allele. Simulations were carried out as described previously [18], taking the observed allele frequency in Hap Map CEU individuals [52] as the starting frequency, and assuming an effective human population size N_e_ of 10,000 of constant size and a generation time of 20 years.

## Supporting Information

Figure S1 NCO gene conversion frequency per SNP shown for each man assayed at hotspot F. Assays with selector sites in phase with the parental haplotype carrying F6.1A are shown in red, with opposite phase recombinants shown in blue. Details of marker phasing are provided Table S3.
(TIFF)

Figure S2 NCO gene conversion frequency per SNP shown for each man heterozygous at marker K7.4 at hotspot K. Assays with selector sites in phase with the parental haplotype carrying K7.4T are shown in red, with opposite phase recombinants shown in blue. Details of marker phasing are provided in Table S3.
(TIFF)

Figure S3 NCO gene conversion frequency per SNP shown for each man homozygous at marker K7.4 at hotspot K. The two orientations of recombination are shown in different colours for each man.
(TIFF)

Table S1 Summary details of the six hotspots chosen for analysis.
(PDF)

Table S2 Transmission data for initial screening of the six hotspots.
(PDF)

Table S3 *PRDM9* genotypes of men analysed at hotspots F and K, plus selector primers for allele-specific PCR and phasing of heterozygous markers.
(PDF)

Table S4 Comparison of existing limited CO data-sets for hotspots F and K with the expanded data-sets generated in this study.
(PDF)

Table S5 PCR conditions for recombination assays at hotspots E, F, H, K, T & 5A.
(PDF)

Table S6 Oligonucleotides used for recombinant detection by allele-specific hybridisation at hotpots F and K.
(PDF)
