# Supplementary figures and images for: Transmission Distortion Affecting Human Noncrossover but Not Crossover Recombination: A Hidden Source of Meiotic Drive
Source: PLoS Genet. 2014 Feb 6;10(2):e1004106. doi: 10.1371/journal.pgen.1004106 (PMC3916235; doi:10.1371/journal.pgen.1004106)

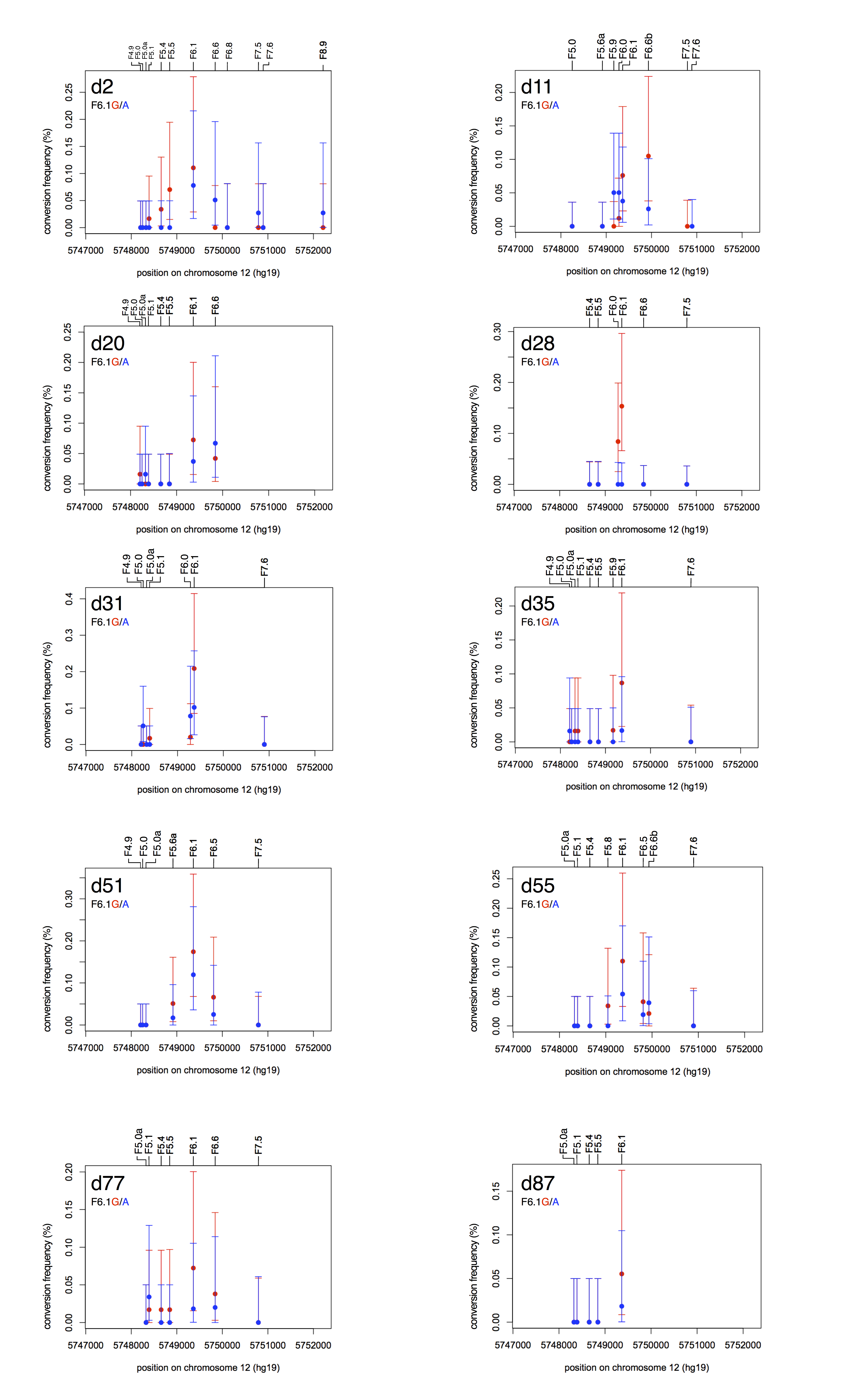

Supplement: Figure S1 — NCO gene conversion frequency per SNP shown for each man assayed at hotspot F. Assays with selector sites in phase with the parental haplotype carrying F6.1A are shown in red, with opposite phase recombinants shown in blue. Details of marker phasing are provided Table S3. (TIFF) [file pgen.1004106.s001.tiff]

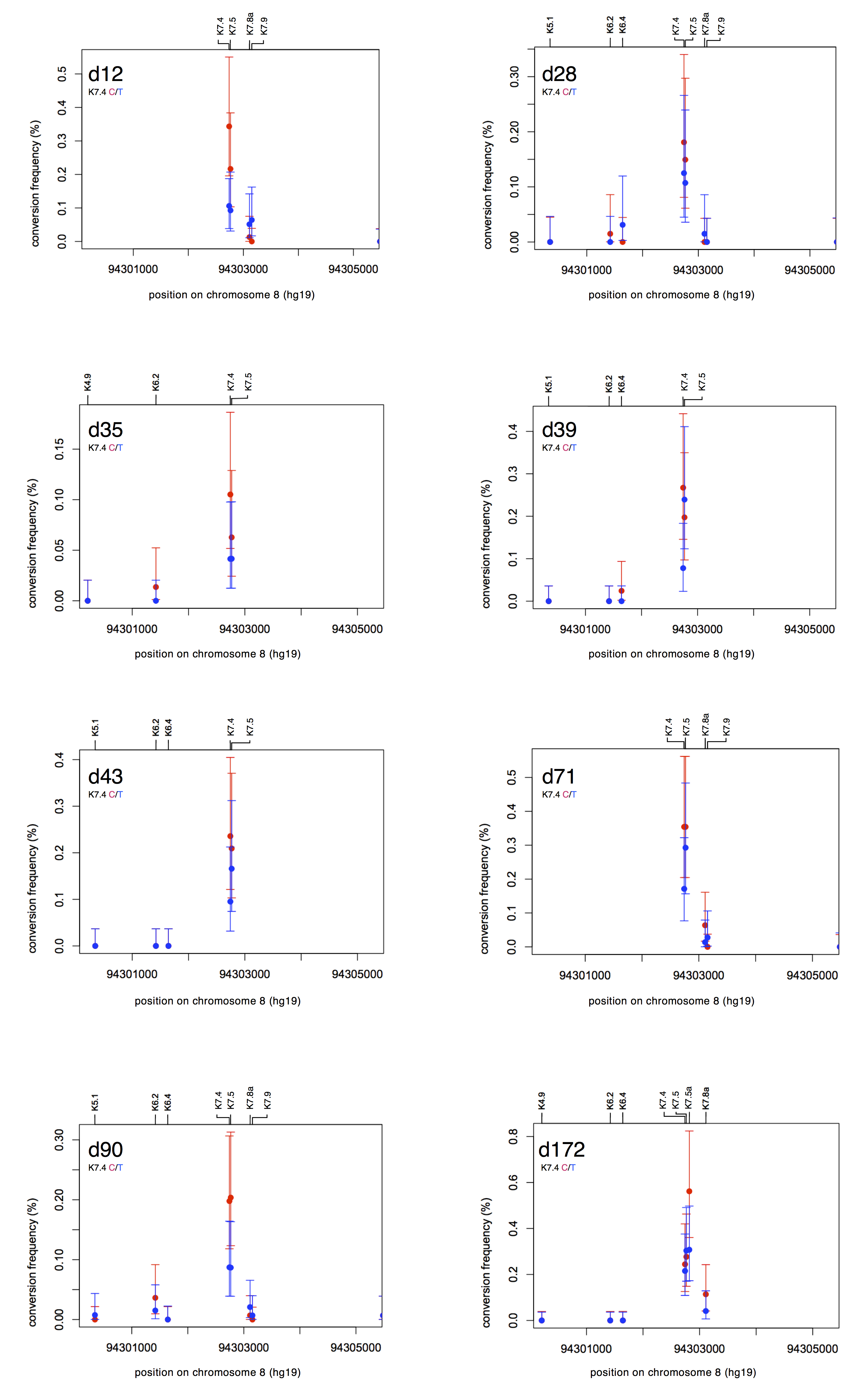

Supplement: Figure S2 — NCO gene conversion frequency per SNP shown for each man heterozygous at marker K7.4 at hotspot K. Assays with selector sites in phase with the parental haplotype carrying K7.4T are shown in red, with opposite phase recombinants shown in blue. Details of marker phasing are provided in Table S3. (TIFF) [file pgen.1004106.s002.tiff]

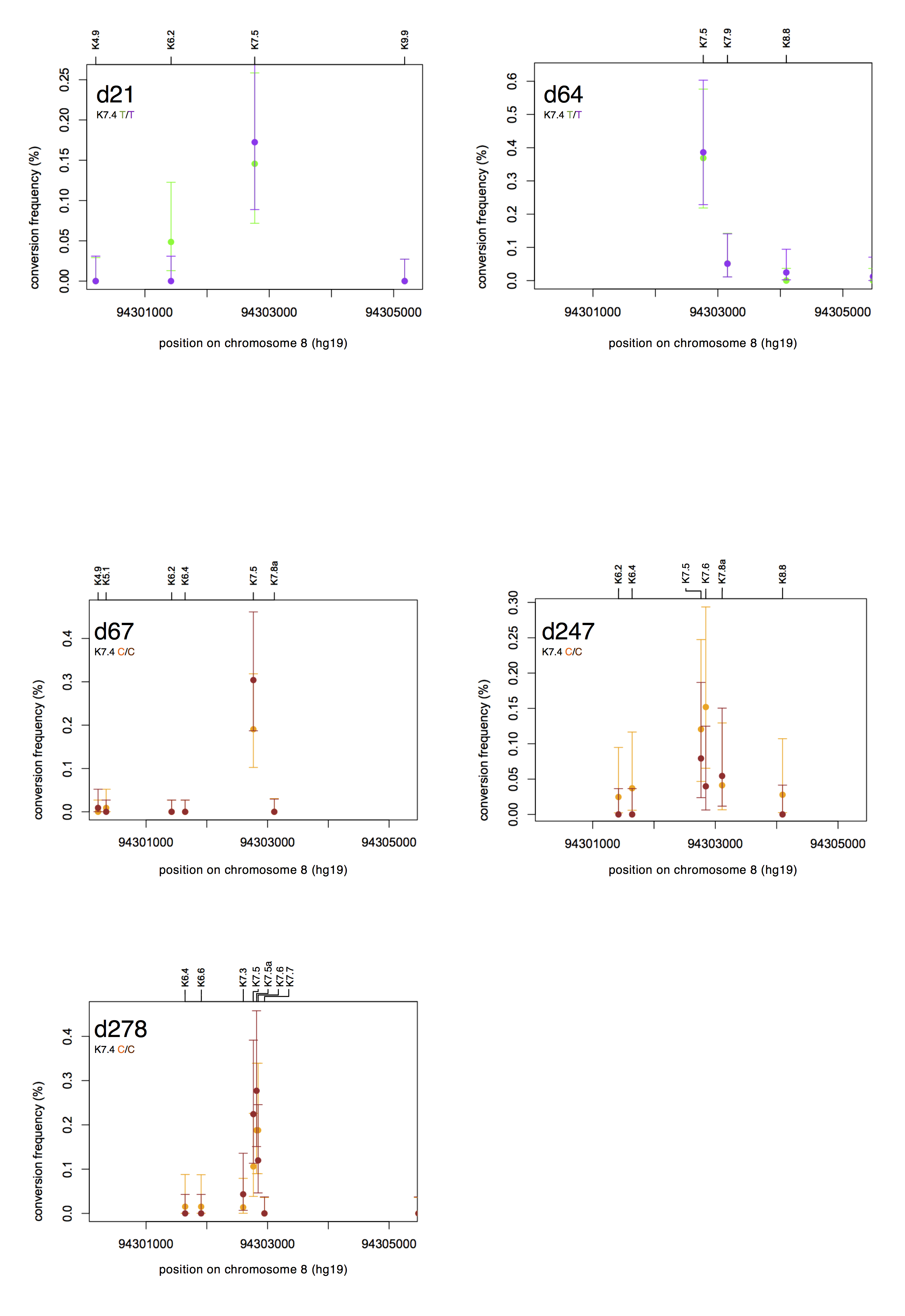

Supplement: Figure S3 — NCO gene conversion frequency per SNP shown for each man homozygous at marker K7.4 at hotspot K. The two orientations of recombination are shown in different colours for each man. (TIFF) [file pgen.1004106.s003.tiff]
