# Supplementary material for: Transmission Distortion Affecting Human Noncrossover but Not Crossover Recombination: A Hidden Source of Meiotic Drive
Source: PLoS Genet. 2014 Feb 6;10(2):e1004106. doi: 10.1371/journal.pgen.1004106 (PMC3916235; doi:10.1371/journal.pgen.1004106)
Supplement: Table S1 — Summary details of the six hotspots chosen for analysis. (PDF) [file pgen.1004106.s004.pdf]

Table S1. Summary details of the six hotspots chosen for analysis

Hotspot:	E	F	H	K	T	5A
**centre location (GRCh37/hg19)**	chr8:137,524,198	chr12:5,749,255	chr3:14,817,625	chr8:94,302,762	chr3:2,426,642	chr5:1,935,700
**genomic context**	intergenic	intronic (ANO2)	Intergenic	intergenic	intronic (CNTN4)	intergenic
**main activating *PRDM9* variant**	A	A	A	A	A	C
**CO frequency per sperm (%)***	0.49	0.97	0.13	0.22	1.10	0.54
**peak frequency (cM/Mb)**	550	1100	180	260	1200	600
**95% width (kb)**	1.4	1.4	1.2	1.4	1.5	1.5
**Informative SNPs ±150 bp from centre (local name) [distance (bp) ; MAF†]**	rs2255417 (E6.8) [47 ; 0.372]	rs11063791 (F5.9) [87 ; 0.346]	rs853334 (H7.5) [-29 ; 0.410]	rs1374633 (K7.4) [20 ; 0.302]	rs9854419 (T7.9) [10 ; 0.346]	rs13355978 (5A7.1) [127 ; 0.238]
		rs386440 (F6.0) [-26 ; 0.277]	rs3899614 (H7.6) [-102 ; 0.457]	rs1374632 (K7.5) [-4 ; 0.406]		rs116141470 (5A7.2) [21 ; 0.169]
		rs10492181 (F6.1) [-108 ; 0.133]				

***** in men with two activating *PRDM9* alleles

† minor allele frequencies as determined from 94 north Europeans for hotspots E, F, H, K & T [refs 37,5], and from 74 Africans for hotspot 5A [ref 9].
