# Supplementary material for: Transmission Distortion Affecting Human Noncrossover but Not Crossover Recombination: A Hidden Source of Meiotic Drive
Source: PLoS Genet. 2014 Feb 6;10(2):e1004106. doi: 10.1371/journal.pgen.1004106 (PMC3916235; doi:10.1371/journal.pgen.1004106)
Supplement: Table S2 — Transmission data for initial screening of the six hotspots. (PDF) [file pgen.1004106.s005.pdf]

Table S2. Transmission data for initial screening of the six hotspots

Hotspot E		E6.8A/G					
		CO			NCO		
man	molecules screened	A	G	% G	A	G	% G
17	14720	20	16	44	9	12	57
56	22080	17	14	45	3	10	77
	TOTAL	**37**	**30**	**45 (ns)**	**12**	**22**	**65 (ns)**

Hotspot F		F5.9T/C						F6.0T/A						F6.1A/G					
		CO			NCO			CO			NCO			CO			NCO		
man	molecules screened	T	C	% C	T	C	% C	A	T	% T	A	T	% T	A	G	% G	A	G	% G
28	16660	n/a	n/a	n/a	n/a	n/a	n/a	81	67	45	8	6	43	83	65	44	0	11	100
11	16560	24	20	45	0	3	100	18	26	59	3	1	25	18	26	59	3	6	67
	TOTAL	**24**	**20**	**45 (ns)**	**0**	**3**	**100 (ns)**	**99**	**93**	**48 (ns)**	**11**	**7**	**61 (ns)**	**101**	**91**	**47 (ns)**	**3**	**17**	**85 (**)**

Hotspot H		H7.5A/G						H7.6A/G					
		CO			NCO			CO			NCO		
man	molecules screened	A	G	% G	A	G	% G	A	G	% G	A	G	% G
25	24840	13	18	58	3	7	70	9	22	71	4	6	60
52	22080	28	21	43	3	5	63	23	26	53	2	4	67
	TOTAL	**41**	**39**	**49(ns)**	**6**	**12**	**67 (ns)**	**34**	**41**	**60 (ns)**	**6**	**10**	**63 (ns)**

Hotspot K		K7.4T/C						K7.5C/G					
		CO			NCO			CO			NCO		
man	molecules screened	T	C	% C	T	C	% C	C	G	% G	C	G	% G
28	13800	14	6	30	8	12	60	12	8	40	7	10	59
90	29440	26	35	57	12	28	70	31	27	47	28	12	30
	TOTAL	**40**	**41**	**51 (ns)**	**20**	**40**	**67 (*)**	**43**	**35**	**45 (ns)**	**35**	**22**	**39 (ns)**

Hotspot T		T7.9A/G					
		CO			NCO		
man	molecules screened	A	G	% G	A	G	% G
25	6992	11	20	65	5	10	67
60	9200	0	2	100	0	1	100
	TOTAL	**11**	**22**	**67 (ns)**	**5**	**11**	**69 (ns)**

Hotspot 5A		5A7.1T/C						5A7.2A/G					
		CO			NCO			CO			NCO		
man	molecules screened	T	C	% C	T	C	% C	A	G	% G	A	G	% G
243	16560	20	30	60	2	2	50	n/a	n/a	n/a	n/a	n/a	n/a
253	15180	n/a	n/a	n/a	n/a	n/a	n/a	39	78	67	6	28	82
	TOTAL	**20**	**30**	**60 (ns)**	**2**	**2**	**50 (ns)**	**39**	**78**	**67 (***)**	**6**	**28**	**82 (***)**

Deviation from expected 50:50 ratio of alleles determined by 2-tailed exact binomial test; (ns) = P>0.05, (*) = P<0.05, (**) = P <0.005, (***) = P<0.0005

n/a, not applicable
