# Supplementary material for: Transmission Distortion Affecting Human Noncrossover but Not Crossover Recombination: A Hidden Source of Meiotic Drive
Source: PLoS Genet. 2014 Feb 6;10(2):e1004106. doi: 10.1371/journal.pgen.1004106 (PMC3916235; doi:10.1371/journal.pgen.1004106)
Supplement: Table S3 — PRDM9 genotypes of men analysed at hotspots F and K, plus selector primers for allele-specific PCR and phasing of heterozygous markers. (PDF) [file pgen.1004106.s006.pdf]

**Table S3. *PRDM9* genotypes of men analysed at hotspots F and K, plus selector primers for allele-specific PCR and phasing of heterozygous markers**

**Hotspot F**

| man | *PRDM9* genotype | 1° selector primers | 2° selector primers | Phasing of heterozygous markers* |
|---|---|---|---|---|
| 2 | A A | F4.6FT / FC | F4.8FC / FT | F4.6T-F4.8C-F4.9G-F5.0T-F5.0aA-F5.1T-F5.4C-F5.5A-F6.1G-F6.6G-F6.8C-F7.5C-F7.6G-F8.9T-F9.2G-F9.4A |
| 11 | A A | F3.8aFG2 / FA2 | F3.9FC / FT | F3.8aG-F3.9T-F5.0T-F5.6aC-F5.9T-F6.0T-F6.1G-F6.6bG-F7.5C |
| 20 | A A | F4.6FT / FC | F4.8FC / FT | F4.6C-F4.8T-F4.9A-F5.0G-F5.0aG-F5.1C-F5.4T-F5.5T-F6.1G-F6.6A-F6.6bG |
| 28 | A A | F13.3RC / RT | F13.0RT2 / RG2 | F5.4T-F5.5T-F6.0T-F6.1G-F6.6G-F7.5T-F9.2A-F10.6A-F11.3C-F12.0C-F13.0T-F13.3C |
| 31 | A A | F4.6FT / FC | F4.8FC / FT | F4.6C-F4.8T-F4.9A-F5.0G-F5.0aG-F5.1C-F6.0A-F6.1G-F7.6C-F9.4G |
| 35 | A A | F4.6FT / FC | F4.8FC / FT | F4.6C-F4.8T-F4.9A-F5.0G-F5.0aG-F5.1C-F5.4T-F5.5T-F5.9C-F6.1G-F7.6C-F9.4G |
| 51 | A A | F4.6FT / FC | F4.8FC / FT | F4.6T-F4.8C-F4.9G-F5.0T-F5.0aA-F5.6aC-F6.1G-F6.5T-F7.5T-F8.1C |
| 55 | A A | F4.6FT / FC | F4.8FC / FT | F4.6C-F4.8T-F5.0aG-F5.1C-F5.4T-F5.8G-F6.1G-F6.5C-F6.6bA-F7.6G-F9.4A |
| 77 | A B | F4.6FT / FC | F4.8FC / FT | F4.6C-F4.8T-F5.0aG-F5.1C-F5.4T-F5.5T-F6.1G-F6.6G-F7.5T-F9.2A |
| 87 | A E | F4.6FT / FC | F4.8FC / FT | F4.6C-F4.8T-F5.0aG-F5.1C-F5.4T-F5.5T-F6.1G-F7.1G |

**Hotspot K**

| man | *PRDM9* genotype | 1° selector primers | 2° selector primers | Phasing of heterozygous markers* |
|---|---|---|---|---|
| 12 | A B | K11.7RC / RT | K10.9RA / RG | K4.9T-K7.4C-K7.5C-K7.8aT-K7.9T-K10.2G-K10.9G-K11.7T |
| 21 | A A | K11.7RC / RT | K10.9RA / RG | K4.9T-K6.2C-K7.5C-K9.9G-K10.2G-K10.9G-K11.7T |
| 28 | A A | K11.7RC / RT | K10.9RA / RG | K4.9C-K5.1G-K6.2T-K6.4T-K7.4C-K7.5G-K7.8aC-K7.9C-K10.2A-K10.9A-K11.7C |
| 35 | A A | K2.9FC / FT2 | K3.2FC2 / FG2 | K2.9T-K3.2G-K4.9T-K6.2C-K7.4C-K7.5C-K9.9G |
| 39 | A A | K2.9FC / FT2 | K3.2FC2 / FG2 | K2.9T-K3.2G-K5.1A-K6.2C-K6.4C-K7.4C-K7.5C-K9.9A |
| 43 | A L20 | K2.9FC / FT2 | K3.2FC2 / FG2 | K2.9C-K3.2C-K4.9C-K5.1G-K6.2T-K6.4T-K7.4C-K7.5C-K10.2A-K10.9A-K11.7C |
| 64 | A A | K11.7RC / RT | K10.9RA / RG | K5.1G-K7.5G-K7.9T-K8.8C-K10.2G-K10.9G-K11.7T |
| 67 | A A | K2.9FC / FT2 | K3.2FC2 / FG2 | K2.9T-K3.2G-K4.9T-K5.1A-K6.2C-K6.4C-K7.5G-K7.8aC-K7.9C |
| 71 | A A | K11.7RC / RT | K10.9RA / RG | K4.9T-K7.4C-K7.5C-K7.8aT-K7.9T-K10.2G-K10.9G-K11.7T |
| 90 | A A | K11.7RC / RT | K10.9RA / RG | K4.9T-K5.1A-K6.2C-K6.4C-K7.4C-K7.5C-K7.8aT-K7.9T-K10.2G-K10.2G-K11.7T |
| 172 | A A | K2.9FC / FT2 | K3.2FC2 / FG2 | K2.9T-K3.2G-K4.9T-K6.2C-K6.4C-K7.4C-K7.5G-K7.5aA-K7.8aC-K8.8T |
| 247 | A A | K2.9FC / FT2 | K3.2FC2 / FG2 | K2.9C-K3.2C-K6.2T-K6.4T-K7.5C-K7.6T- K7.8aT-K8.8T-K10.2G-K10.9G-K11.7T |
| 278 | A A | K11.7RC / RT | K10.9RA / RG | K6.2T-K6.4T-K6.6C-K7.3G-K7.5G-K7.5aT-K7.6T-K7.7(-)-K10.2G-K10.9G-K11.7T |

* For hotspot F haplotypes bearing F6.1G are shown. For K7.4 heterozygotes, haplotypes with K7.4C are shown.
