# Supplementary material for: Transmission Distortion Affecting Human Noncrossover but Not Crossover Recombination: A Hidden Source of Meiotic Drive
Source: PLoS Genet. 2014 Feb 6;10(2):e1004106. doi: 10.1371/journal.pgen.1004106 (PMC3916235; doi:10.1371/journal.pgen.1004106)
Supplement: Table S4 — Comparison of existing limited CO data-sets for hotspots F and K with the expanded data-sets generated in this study. (PDF) [file pgen.1004106.s007.pdf]

Table S4. Comparison of existing limited CO data-sets for hotspots F and K with the expanded data-sets generated in this study

**Hotspot F**	**Previous analyses**	**Present study**
number of men analysed	3	10
mean crossover frequency, %	0.97	0.81
CO frequency range, %	0.82-1.09	0.21-1.30
mean centre location*	chr12:5,749,255	chr12:5,749,271
mean 95% width (kb)	1.40	1.60
**Hotspot K**	**Previous analyses**	**Present study**
number of men analysed	3	13
mean crossover frequency, %	0.22	0.26
CO frequency range, %	0.19-0.26	0.04-0.44
mean centre location*	chr8:94,302,762	chr8:94,302,727
mean 95% width (kb)	1.40	1.46

* Given with respect to February 2009 assembly (GRCh37/hg19)
