# Supplementary material for: Transmission Distortion Affecting Human Noncrossover but Not Crossover Recombination: A Hidden Source of Meiotic Drive
Source: PLoS Genet. 2014 Feb 6;10(2):e1004106. doi: 10.1371/journal.pgen.1004106 (PMC3916235; doi:10.1371/journal.pgen.1004106)
Supplement: Table S5 — PCR conditions for recombination assays at hotspots E, F, H, K, T & 5A. (PDF) [file pgen.1004106.s008.pdf]

Table S5. PCR conditions for recombination assays at hotspots E, F, H, K, T and 5A

Hotspot E

**primer**	**sequence 5'-3'**	**PCR conditions**
**1° PCR:**		
E12.4R+2 or	ATTGACCCAGTTTACTACACATAGT	25 cycles: 96°C 20 sec, 58°C, 30 sec, 65°C 9 min
E12.4R-	ACCCAGTTTACTACACAGT	25 cycles: 96°C 20 sec, 58°C, 30 sec, 65°C 9 min
with E4.9F	GAGTATGAATGGTGTCTCCC	
**2° PCR:**		
E10.6RT or	CAGCCTGGTCAACCTGA	38 cycles: 96°C 20 sec, 62°C, 30 sec, 65°C 6 min
E10.6RC	AGCCTGGTCAACCTGG	38 cycles: 96°C 20 sec, 62°C, 30 sec, 65°C 6 min
with E5.6F	TAACCATGCACTGAGCTCCC	

Hotspot F

**primer**	**sequence 5'-3'**	**PCR conditions**
**1° PCR:**		
F4.6FT or	AGGGGATCTGGCTGCCT	26 cycles: 96°C 20 sec, 63°C 30 sec, 65°C 7 min
F4.6FC	AGGGGATCTGGCTGCCC	26 cycles: 96°C 20 sec, 63°C 30 sec, 65°C 7 min
with F9.7R	ATCCAAATGGCCAATGAGCC	
**2° PCR:**		
F4.8FC or	GTCCAGAGTCCTACTTGAC	6 cycles: 96°C 20 sec, 65°C 30 sec, 65°C 6 min; 6 cycles: 96°C 20 sec, 64°C 30 sec, 65°C 6 min; 21 cycles: 96°C 20 sec, 63°C 30 sec, 65°C 6 min
F4.8FT	GTCCAGAGTCCTACTTGAT	6 cycles: 96°C 20 sec, 63°C 30 sec, 65°C 6 min; 6 cycles: 96°C 20 sec, 62°C 30 sec, 65°C 6 min; 21 cycles: 96°C 20 sec, 61°C 30 sec, 65°C 6 min
with 9.5R	AGTACATATATCCTGGGGCC	
**1° PCR:**		
F3.8aFG or	GAGYACCAACCCAGTCACG	25 cycles: 96°C 20 sec, 64°C 30 sec, 65°C 9 min
F3.8aFA	GAGYACCAACCCAGTCACA	25 cycles: 96°C 20 sec, 62°C 30 sec, 65°C 9 min
with F9.7R	ATCCAAATGGCCAATGAGCC	
**2° PCR:**		
F3.8FT or	ccccCAATGCTACTCTTCTGGAT	35 cycles: 96°C 20 sec, 60°C 30 sec, 65°C 7 min
F3.8FC	ccccCAATGCTACTCTTCTGGAC	10 cycles: 96°C 20 sec, 65°C 30 sec, 67°C 7 min 25 cycles: 96°C 20 sec, 64°C 30 sec, 65°C 7 min
with 9.5R	AGTACATATATCCTGGGGCC	
**1° PCR:**		
F13.3RC or	CGTTTGTGGATAGACCAGG	25 cycles: 96°C 20 sec, 63°C 30 sec, 65°C 11 min
F13.3RT	CGTTTGTGGATAGACCAGA	25 cycles: 96°C 20 sec, 63°C 30 sec, 65°C 11 min
with F3.5F	CAACTCCAGACCTACTGACC	
**2° PCR:**		
F13.0RG or	GCCAGCATGGGCAAGACC	35 cycles: 96°C 20 sec, 63°C 30 sec, 65°C 10 min
F13.0RT	TGCCAGCATGGGCAAGACA	35 cycles: 96°C 20 sec, 63°C 30 sec, 65°C 10 min
with F4.0F	ATTGATCCAAGGTCTGGAGG	

Hotspot H

**primer**	**sequence 5'-3'**	**PCR conditions**
**1° PCR:**		
H2.0FA or	TCCCAGAGCTACCTCAAA	25 cycles: 96°C 20 sec, 61°C, 30 sec, 65°C 11 min
H2.0FG	TCCCAGAGCTACCTCAAG	25 cycles: 96°C 20 sec, 62°C, 30 sec, 65°C 11 min
with H12.3R	ATACACGGCAGGCTTTGCCC	
**2° PCR:**		
H2.5FA or	GCTCCAGAAGGACCCTCA	35 cycles: 96°C 20 sec, 64°C, 30 sec, 65°C 9 min
H2.5FG	CTCCAGAAGGACCCTCG	35 cycles: 96°C 20 sec, 61°C, 30 sec, 65°C 9 min
with H10.3R	ATCTCTACTCCTACCCCTAC	

Hotspot K

**primer**	**sequence 5'-3'**	**PCR conditions**
**1° PCR:**		
K11.7RC or	TGAAGGTAGAATTGGCCG	25 cycles: 95°C 20 sec, 59.5°C 30 sec, 65°C 9 min
K11.7RT	GTGAAGGTAGAATTGGCCA	25 cycles: 95°C 20 sec, 60°C 30 sec, 65°C 9 min
with K4.4F	CACATGTCAGCACACATTGCCC	
**2° PCR:**		
K10.9RA or	CCCCCCCCCTTAATTTTTAAACT	38 cycles: 95°C 20 sec, 60°C 30 sec, 65°C 8 min
K10.9RG	CCCCCCCCCTTAATTTTTAAACC	38 cycles: 95°C 20 sec, 60°C 30 sec, 65°C 8 min
with K4.5F	GCGAGGAGTCCTAAACTCCC	
**1° PCR:**		
K2.9FC	CCAGGTCCCCTGTCTC	28 cycles: 95°C 20 sec, 62°C 30 sec, 65°C 9 min
K2.9FT2	GTTCCAGGTCCCCTGTCTT	25 cycles: 95°C 20 sec, 63°C 30 sec, 65°C 9 min
with K10.8R	CTTGGCTGAAGCCTCTCCC	

2° PCR:

K3.2FC2 or	ccccGAAAAACAGCACATCAGAC	10 cycles: 95°C 20 sec, 61°C 30 sec, 65°C 9 min; 25 cycles: 95°C 20 sec, 60°C 30 sec, 65°C 9 min
K3.2FG2	ccccGAAAAACAGCACATCAGAG	10 cycles: 95°C 20 sec, 61°C 30 sec, 65°C 9 min; 25 cycles: 95°C 20 sec, 60°C 30 sec, 65°C 9 min
with K10.8R	CTTGGCTGAAGCCTCTCCC	

Hotspot T

**primer**	**sequence 5'-3'**	**PCR conditions**
**1° PCR:**		
T10.8RC or	ccccCTTTGTTTTCTGTCTTCTG	25 cycles: 96°C 20 sec, 62°C, 30 sec, 65°C 11 min
T10.8RT	ccccCTTTGTTTTCTGTCTTCTA	25 cycles: 96°C 20 sec, 58°C, 30 sec, 65°C 11 min
with T1.1F	GGCCCAACATACATTGGCTC	
**2° PCR:**		
T9.6+ or	ccccGCAATTTTCATGTATCTC	35 cycles: 96°C 20 sec, 58°C, 30 sec, 65°C 9.5 min
T9.6-	ccccTGCAATTTTCATGTACTC	35 cycles: 96°C 20 sec, 58°C, 30 sec, 65°C 9.5 min
with T1.3F	GAGGACAGTCCTGGCTGTG	

Hotspot 5A

**primer**	**sequence 5'-3'**	**PCR conditions**
**1° PCR:**		
5A9.9aRG or	CTTGTGATCTCACAGCAC	25 cycles: 96°C 20 sec, 55°C, 30 sec, 65°C 7.5 min
5A9.9aRC	CTTGTGATCTCACAGCAG	25 cycles: 96°C 20 sec, 55°C, 30 sec, 65°C 7.5 min
with 5A3.6F	AGCTGACATGGCCAAGTTCC	
**2° PCR:**		
5A9.9RC or	TCTCACAGCACAGGTTGG	32 cycles: 96°C 20 sec, 58°C, 30 sec, 65°C 7.5 min
5A9.9RG	TCTCACAGCAGAGGTTGC	32 cycles: 96°C 20 sec, 55°C, 30 sec, 65°C 7.5 min
with 5A3.7F	CCTGGCGAATAGCTCTTTCC	
