# Supplementary material for: Transmission Distortion Affecting Human Noncrossover but Not Crossover Recombination: A Hidden Source of Meiotic Drive
Source: PLoS Genet. 2014 Feb 6;10(2):e1004106. doi: 10.1371/journal.pgen.1004106 (PMC3916235; doi:10.1371/journal.pgen.1004106)
Supplement: Table S6 — Oligonucleotides used for recombinant detection by allele-specific hybridisation at hotpots F and K. (PDF) [file pgen.1004106.s009.pdf]

**Table S6. Oligonucleotides used for recombinant detection by allele-specific hybridisation at hotpots F and K**

**Hotspot F**

| | | Allele-Specific Oligonucleotides | | |
|---|---|---|---|---|
| **rs number** | **chr12(hg19)** | **allele** | **probe name** | **sequence 5'-3'** |
| rs11063787 | 5748205 | G | F4.9G | AGCTTCTGGTATGCTCTT |
| " | " | A | F4.9A | AGCTTCTAGTATGCTCTT |
| rs11063788 | 5748248 | T | F5.0T | GAGAAAACTCCACAGAAA |
| " | " | G | F5.0G | GAGAAAACGCCACAGAAA |
| rs11063789 | 5748324 | A | F5.0aA | CTTTCCAGAAAAGGGAAG |
| " | " | G | F5.0aG | CTTTCCAGGAAAGGGAAG |
| rs1558780 | 5748391 | C | F5.1C | CATGGCACAYGATAGTAG |
| " | " | T | F5.1T | CATGGCATAYGATAGTAG |
| rs10849308 | 5748656 | T | F5.4T2 | CGTGCTGAAGGTACTACT |
| " | " | C | F5.4C2 | CGTGCTGGAGGTACTACT |
| rs365211 | 5748844 | T | F5.5T2 | CCACTCCACCTGTCCCC |
| " | " | A | F5.5A2 | CCACTCCTCCTGTCCCC |
| rs365131 | 5748918 | C | F5.6aC | CCTCAGGCGATGGGAAGC |
| " | " | A | F5.6aA | CCTCAGGAGATGGGAAGC |
| rs393861 | 5749047 | G | F5.8G | GATGAGAGTCCAGGCTGT |
| " | " | A | F5.8A | GATGAGAATCCAGGCTGT |
| rs11063791 | 5749168 | T | F5.9T | TCGCCTCTGACAATTCCC |
| " | " | C | F5.9C | TCGCCTCCGACAATTCCC |
| rs386440 | 5749281 | A | F6.0A2 | CTTGTGTTAGAAACCAGC |
| " | " | T | F6.0T2 | CTTGTGTAAGAAACCAGC |
| rs10492181 | 5749363 | A | F6.1A2 | TGCCAATCTACCGAGTCA |
| " | " | G | F6.1G2 | TGCCAATCCACCGAGTCA |
| rs9795821 | 5749807 | T | F6.5T | TCACTGGTCATCAGAGAA |
| " | " | C | F6.5C | TCACTGGCCATCAGAGAA |
| rs384401 | 5749841 | G | F6.6G2 | GGGATGGTACCTCATTGT |
| " | " | A | F6.6A2 | GGGATGGTATCTCATTGT |
| rs11063792 | 5749932 | A | F6.6bA | CTTTTACACTGTTGGTGG |
| " | " | G | F6.6bG | CTTTTACGCTGTTGGTGG |
| rs111733683 | 5750109 | T | F6.8T | GCAGCACTATTCACAACA |
| " | " | C | F6.8C | GCAGCACCATTCACAACA |
| rs374793 | 5750357 | A | F7.1A | TGGACACAGGGCGAGGAA |
| " | " | G | F7.1G | TGGACACGGGGCGAGGAA |
| rs417672 | 5750791 | T | F7.5T2 | GAATGGGGAGAGCCCC |
| " | " | C | F7.5C2 | GAATGGGGGGAGCCCC |
| rs1558506 | 5750896 | G | F7.6G | TCAATTAGTTATTGGTTC |
| " | " | C | F7.6C | TCAATTACTTATTGGTTC |
| rs2110166 | 5751342 | C | F8.1C | CAATTCTCTTTTCTGTCT |
| " | " | T | F8.1T | CAATTCTTTTTTCTGTCT |
| rs12811402 | 5752212 | T | F8.9T | GTGAACATGCAGAAAAAT |
| " | " | C | F8.9C | GTGAACACGCAGAAAAAT |

**Hotspot K**

| rs number | chr8(hg19) | allele | probe name | sequence 5'-3' |
|---|---|---|---|---|
| | | | **Allele-Specific Oligonucleotides** | |
| rs6999442 | 94300209 | T | K4.9T | GTTCAATACATTACTCTG |
| " | " | C | K4.9C | GTTCAATGCATTACTCTG |
| rs35234504 | 94300343 | G | K5.1G | TCTGGCTCATGGGTCACC |
| " | " | A | K5.1A | TCTGGCTTATGGGTCACC |
| rs2034306 | 94301420 | T | K6.2T | AAGACATTTTGGACTTCT |
| " | " | C | K6.2C | AAGACATCTTGGACTTCT |
| rs7005566 | 94301642 | T | K6.4T | GTGCCAGACACGTGGTAA |
| " | " | C | K6.4C | GTGCCAGGCACGTGGTAA |
| rs56791026 | 94301906 | C | K6.6C | CTAAAAGCCTTACCAGAT |
| " | " | T | K6.6T | CTAAAAGTCTTACCAGAT |
| rs1374634 | 94302598 | G | K7.3G | ACCAGGCGGGACACCGAC |
| " | " | A | K7.3A | ACCAGGCAGGACACCGAC |
| rs1374633 | 94302742 | C | K7.4C | GAGAGGGCACCCTGCCTA |
| " | " | T | K7.4T | GAGAGGGTACCCTGCCTA |
| rs1374632 | 94302766 | G | K7.5G | TGTGATGCCCTCCCTGAT |
| " | " | C | K7.5C | TGTGATGGCCTCCCTGAT |
| rs7814351 | 94302818 | T | K7.5aT | TCATGGTTTTCACCTAAA |
| " | " | A | K7.5aA | TCATGGTATTCACCTAAA |
| rs6471366 | 94302844 | T | K7.6T | TAACCTTTGCAATAGTCC |
| " | " | C | K7.6C | TAACCTTCGCAATAGTCC |
| rs10716160 | 94302948 | + | K7.7 + | AACCTCTAGCCTCCTGGG |
| " | " | - | K7.7 - | AACCTCTGCCTCCTGGGT |
| rs10088843 | 94303110 | C | K7.8aC | ATTCACCCGCCTTGGCCT |
| " | " | T | K7.8aT | ATTCACCTGCCTTGGCCT |
| rs6471367 | 94303153 | T | K7.9T | GAGCCACTGTRCCCGGCC |
| " | " | C | K7.9C | GAGCCACCGTRCCCGGCC |
| rs9918898 | 94304095 | T | K8.8T | CCAACTTTTGTGAGCCTC |
| " | " | C | K8.8C | CCAACTTCTGTGAGCCTC |
| rs1550883 | 94305178 | A | K9.9A | TTCATGCGTCTCAGTCAC |
| " | " | G | K9.9G | TTCATGCATCTCAGTCAC |
| rs1550882 | 94305482 | A | K10.2A | TGGGTCAATTTCACAGAT |
| " | " | G | K10.2G | TGGGTCAGTTTCACAGAT |
| rs7829351 | 94306209 | A | K10.9A | GTGTGTCAGTTTAAAAAT |
| " | " | G | K10.9G | GTGTGTCGGTTTAAAAAT |
